# Ascorbate as a Co-Factor for Fe- and 2-Oxoglutarate Dependent Dioxygenases: Physiological Activity in Tumor Growth and Progression

**DOI:** 10.3389/fonc.2014.00359

**Published:** 2014-12-10

**Authors:** Caroline Kuiper, Margreet C. M. Vissers

**Affiliations:** ^1^Department of Pathology, Centre for Free Radical Research, University of Otago, Christchurch, New Zealand

**Keywords:** ascorbate, cancer, hydroxylation, hypoxia-inducible factor-1, TET enzymes, tumor microenvironment, vitamin C

## Abstract

Ascorbate is a specific co-factor for a large family of enzymes known as the Fe- and 2-oxoglutarate-dependent dioxygenases. These enzymes are found throughout biology and catalyze the addition of a hydroxyl group to various substrates. The proline hydroxylase that is involved in collagen maturation is well known, but in recent times many new enzymes and functions have been uncovered, including those involved in epigenetic control and hypoxia-inducible factor (HIF) regulation. These discoveries have provided crucial mechanistic insights into how ascorbate may affect tumor biology. In particular, there is growing evidence that HIF-1-dependent tumor progression may be inhibited by increasing tumor ascorbate levels. However, rigorous clinical intervention studies are lacking. This review will explore the physiological role of ascorbate as an enzyme co-factor and how this mechanism relates to cancer biology and treatment. The use of ascorbate in cancer should be informed by clinical studies based on such mechanistic hypotheses.

In 1747, James Lind, a surgeon with the British Royal Navy, performed what is considered the first human clinical trial in an attempt to treat scurvy, administering various potential remedies, such as cider, vinegar, or sea water to a group of 12 scurvy-stricken sailors. The only patients to recover were those who received lemons and oranges, but almost 200 years would pass before the curative compound was identified as hexuronic acid, later named ascorbic acid (ascorbate) for its anti-scorbutic (i.e., anti-scurvy) properties (1). This is commonly known today as vitamin C.

Many of the symptoms of scurvy – gum disease, bleeding, and poor wound healing – are considered to be the result of defective collagen production. It was found that the enzyme responsible for the formation of hydroxy-proline, collagen prolyl-4-hydroxylase (C-P4H), specifically required ascorbate as a co-factor for its activity (2). Hence, under conditions of ascorbate-deficiency, C-P4H loses activity, collagen cannot cross-link sufficiently, and connective tissues can deteriorate.

Collagen prolyl-4-hydroxylase belongs to the family of enzymes known as the Fe- and 2-oxoglutarate dependent dioxygenases (2-OGDDs) that have a wide range of biological functions (3) (Table 1). In mammalian cells, members of this family modify hypoxia-inducible factor (HIF) (4), convert dopamine to noradrenaline, are involved in the α-amidation of numerous pro-hormones (5) and carnitine biosynthesis (6, 7). It has more recently been discovered that 2-OGDDs are epigenetic erasers; these enzymes hydroxylate methyl-lysine residues on histones [Jumonji-C domain-containing histone demethylases (JHDMs)] (8), and ten-eleven translocases (TETs) hydroxylate 5-methyl-cytosine (9). In addition, RNA and ribosomal hydroxylases have very recently been characterized (10, 11).

**Table 1:** **Various members of the 2-oxoglutarate dioxygenase family in mammalian cells, their known substrates, and biological functions**.

Enzyme family/function	Enzyme(s)	Known substrate(s)	Reference
HIF-hydroxylases	PHDs 1–3	HIF-α	(4)
	FIH	HIF-α, ARD proteins	(12)
Cytosine demethylases	TET1–3	5mC	(9, 13)
JMJC histone demethylases	Numerous – JHDMs, KDMs	Methylated histones	(14, 15)
DNA and RNA demethylases	Numerous – AlkB family, FTO	DNA, RNAs, histones	(16, 17)
Ribosomal hydroxylases	MINA53, NO66, OGFOD1	60S ribosomal proteins	(11, 18)
Collagen hydroxylase	C-P4H	Collagen proline residues	(2)
Noradrenalin synthesis	D-βH	Dopamine	(19)
Carnitine synthesis	GBBH	Trimethyl-lysine	(6, 7)
Pro-hormone maturation	PHM	Peptidyl-lysine	(5)

*HIF, hypoxia-inducible factor; PHD, prolyl-hydroxylase domain-containing protein; FIH, factor inhibiting HIF; ARD, ankyrin repeat domain-containing proteins; TET, ten-eleven translocation enzyme; 5mC, 5-methyl-cytosine; JHDM, jumonji histone demethylases; KDM, lysine demethylases; MINA53, Myc-induced nuclear antigen; OGFOD1, 2-oxoglutarate and iron-dependent oxygenase domain-containing 1; FTO, fat-mass and obesity associated protein; C-P4H, collagen prolyl-4-hydroxylase; D-βH, dopamine beta-hydroxylase; GBBH, γ-butyrobetaine dioxygenase; PHM, peptidylglycine α-hydroxylating monooxygenase*.

Clearly, the hydroxylation of diverse proteins, DNA and RNA is proving to be a widespread phenomenon. By the addition of a hydroxyl group to their respective substrates, 2-OGDDs are responsible for altering key protein–protein interactions and nucleic acid structure and function that can result in dramatic effects on cell signaling, gene expression, and tissue function. As ascorbate is a specific co-factor for these enzymes, it will be of great interest to determine how ascorbate availability affects their function and the consequences for tumor biology. The following sections will detail the biochemistry of the 2-OGDDs and the activity of ascorbate as a co-factor, and consider whether this activity has any relevance to tumor progression.

## Fe- and 2-Oxoglutarate-Dependent Dioxygenases

Despite such large substrate diversity, the catalytic cycle of 2-OGDDs is thought to be highly conserved (3). The enzymes are characterized by a core structural motif consisting of eight β-strands arranged in a “jelly roll” or double-stranded β-helix, surrounded by α-helices (20). The active site within the double-stranded β-helix contains a non-haem iron that is coordinated by a “facial triad” of two histidines and one aspartate/glutamate, with the remaining three coordination sites occupied by labile water molecules (20). This is thought to give the catalytic iron a relatively exposed and flexible arrangement compared to haem oxygenases, allowing for a wide range of catalytic oxidations. However, it may also make the active site more prone to auto-oxidation (21).

**Figure 1:**
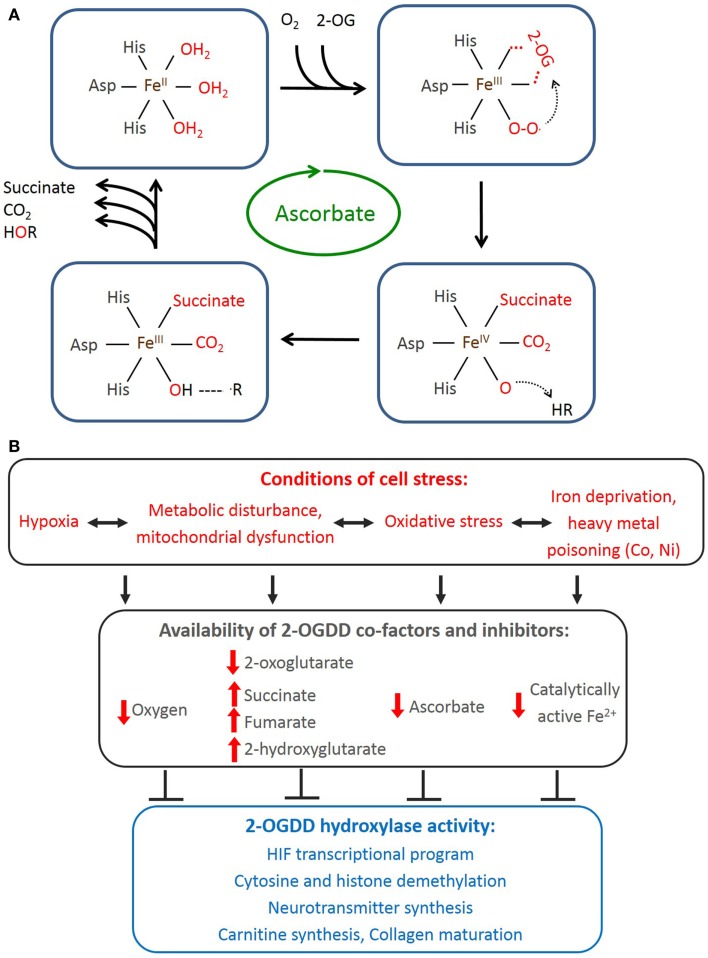
**2-Oxoglutarate-dependent dioxygenase reaction cycle and factors affecting their activity in mammalian cells**. **(A)** Representation of 2-OGDD catalytic cycle. One atom of molecular oxygen is incorporated into the hydroxylated substrate and the other into succinate. 2-OG is converted to succinate, releasing CO_2_. Ferrous iron and ascorbate are specific co-factors for this reaction. **(B)** Cellular stressors deprive 2-OGDDs of their required co-factors resulting in inhibition of multiple potential pathways.

The catalytic cycle (Figure 1A) starts with 2-OG binding, allowing entry of the prime substrate and displacing a water molecule from Fe^2+^. Subsequent binding of molecular O_2_ to Fe^2+^ catalyzes the oxidative decarboxylation of 2-OG to succinate and generates a highly reactive ferryl-oxo species (22) that hydroxylates the prime substrate, regenerating Fe^3+^, which is reverted back to Fe^2+^ followed by release of the substrates and CO_2_. One atom of the O_2_ is incorporated into the hydroxylated substrate and the other into succinate. The CO_2_ is derived from the oxidative decarboxylation of 2-OG to succinate (3, 20). Ascorbate is a necessary co-factor for this reaction, and although its role remains to be determined, it is thought to be required to maintain Fe^2+^ in its reduced state.

Well-characterized members of the 2-OGDD family now include the HIF-hydroxylases (20). These enzymes are thought to have particular relevance to tumor biology due to their direct effects on HIF activation. HIF-1 is a transcription factor known to up-regulate hundreds of genes involved in maintaining oxygen and energy homeostasis under conditions of cell stress, such as hypoxia – a common feature of solid tumors (23). The activation state of HIF-1 is under dual control, with HIF-1α protein stability and transcriptional activity being regulated by hydroxylation reactions. HIF-1α protein is constantly synthesized in most cells in the body, and under normal, physiological conditions is rapidly degraded (24) following hydroxylation of proline residues 402 and 564 by prolyl-hydroxylases (PHD) 1–3 (25, 26) (Table 1). This modification initiates ubiquitination and proteasomal degradation via binding of the tumor-suppressor von Hippel–Lindau protein (pVHL) and an E3 ubiquitin ligase complex (27, 28).

A further hydroxylation event on asparagine 803 of HIF-1α by factor inhibiting HIF (FIH; Table 1) prevents co-activation with p300 and transcriptional activation (29). These events combine to enable a rapid response to cell stressors, where a decrease in hydroxylation activity immediately halts HIF-1α degradation, both allowing the protein to accumulate and also to activate a transcriptional response (20). The activation of HIF-1 is, therefore, dependent upon the activity of the hydroxylases responsible for modification of the HIF-1α subunit.

### 2-OGDD regulation and co-factor dependence

The requirements for the 2-OGDD catalytic cycle (O_2_, Fe^2+^, 2-oxoglutarate, and ascorbate) provide clues as to the physiological conditions that might affect 2-OGDD activity (Figure 1B).

Although the HIF-hydroxylases clearly respond to changes in cellular O_2_ levels and are considered direct cellular oxygen sensors, it is currently unknown whether other 2-OG dioxygenases are similarly sensitive. An example of this has recently been described: in tumorigenic neuroblastoma cells, TET1 was shown to be up-regulated under hypoxia via HIF-1, and to cause increased 5hmC levels specifically at HIF-binding sites, suggesting a synergistic relationship (30). This is somewhat counter-intuitive, as hypoxia would also be expected to inhibit TET activity and 5hmC formation. However, whether the TET catalytic cycle is sensitive to O_2_ changes is yet to be shown. This highlights the complexity of 2-OGDD biology, with each enzyme and isoform having distinct activity dynamics despite sharing a highly conserved active site.

Iron is also a necessary co-factor for 2-OGDD activity, and its substitution/depletion can disrupt HIF-hydroxylase activity and activate the HIF-1 response. Iron chelators such as desferrioxamine (DFO), and Co^2+^ and Ni^2+^ ions robustly induce HIF-1, and this is thought to be through poisoning of the hydroxylases via removal of enzyme-bound iron (4, 26, 31). The apparent *K_m_* values for Fe^2+^ are low, with 0.03 μM for PHD1 and 2, 0.1 μM for PHD3 (32) and 0.5 μM for FIH (33). This suggests tight binding of Fe^2+^ to the HIF-hydroxylase active site, despite the coordination chemistry predicting a labile arrangement (21). However, whether Fe^3+^ produced during enzymatic cycling has the same tight binding as Fe^2+^ is unknown.

2-OG (also known as α-ketoglutarate) is an intermediary metabolite of the TCA cycle. Other 2-oxoacids from the TCA cycle, such as succinate and fumarate, can compete with 2-OG to inhibit HIF-hydroxylase activity and induce HIF-1 *in vitro* (34). Pyruvate, oxaloacetate, and malate have also been shown to have similar HIF-1-inducing effects, and may cause significant basal HIF-1 activation under normoxic conditions (35, 36). HIF-1 activity can be increased by mutations in the TCA cycle enzymes succinate dehydrogenase and fumarate dehydrogenase, which cause a build-up of succinate and fumarate, respectively (37). Furthermore, mutated forms of isocitrate dehydrogenase-1 generate 2-hydroxyglutarate (2-HG) that acts as a competitive inhibitor of the HIF-hydroxylases and activates HIF-1 (38, 39). These mutations have the potential to drive non-hypoxic HIF-1 activity and tumorigenesis (40), and have been clinically associated with the susceptibility to renal cancer and paragangliomas (38). The availability of glycolytic and energy intermediates may confer a complex metabolic-sensing role to the HIF-hydroxylases. However, exactly which 2-oxoacids are relevant under physiological conditions, and to what degree, is unknown.

It has been suggested that the HIF-hydroxylases can be inactivated by production of reactive oxygen species produced by mitochondria in response to hypoxia (41). There is evidence that HIF-hydroxylase activity can be inhibited by H_2_O_2_, inducing HIF-1 in a process requiring functional mitochondria, although the precise mechanisms behind this are unclear (35, 42–44). Conversely, mitochondrial H_2_O_2_ production has been shown to be decreased in response to hypoxia (45) and another study found no association between levels of reactive oxygen species (DCF fluorescence) and HIF-1 target gene expression (46). FIH was shown to be sensitive to H_2_O_2_-mediated inactivation, whereas PHD2 was not, providing an explanation for some of these effects (47), where H_2_O_2_ could influence transcriptional inactivation of HIF-1 without affecting its protein stability.

## Ascorbate as Regulator of 2-OGDD Activity

The *K_m_* values for ascorbate for the HIF-hydroxylases and C-P4H are relatively high [140–300 μM (33, 48, 49)], indicating high intracellular requirements and susceptibility to ascorbate loss. However, the exact role of ascorbate in the hydroxylase reaction is unclear. Most data on ascorbate and hydroxylase activity had come from studies in the 1970–1980s on purified C-P4H in the context of collagen proline-hydroxylation that may also be relevant to other 2-OGDDs (22, 50).

Ascorbate was found to be important, if not essential, for C-P4H activity, with an optimal concentration of 1–2 mM (49, 51–55). In its absence, C-P4H could catalyze only ~15–30 reaction cycles over 8 s before the reaction ceased, and addition of 1 mM ascorbate to the reaction mixture rescued 100% of enzyme activity (2). Another study found C-P4H had a 54% lower initial reaction rate in the absence of ascorbate, which decreased to ~8% of total activity within 30 s with subsequent ascorbate addition able to rescue partial activity (51). Others have found no C-P4H activity without ascorbate, demonstrating an absolute requirement for this co-factor (54, 55). Furthermore, ascorbate was needed for activity prior to 2-OG addition, suggesting that under constant turnover conditions, a permanent ascorbate presence is required (51, 54). Although these results may be influenced by variation in co-substrate concentrations or order of addition, it is clear that ascorbate has a significant effect on hydroxylase activity, with recombinant PHD2 activity having also been shown to increase dose-dependently with ascorbate (56).

Several recent independent studies have demonstrated the specific requirement of ascorbate for TET activity, with a consequent widespread effect on DNA demethylation (57–60). TET enzymes catalyze the conversion of 5mC to 5hmC, which can then be further oxidized and converted to an unmodified (demethylated) cytosine. There was a dose-dependent increase in 5hmC levels of up to fourfold above baseline in ascorbate-treated cells (0–1000 μM) (59, 60), which was mediated by the catalytic domain of TET1 and 2 (60). In addition, the reaction rate of TET2 increased eightfold in the presence of ascorbate (60). Ascorbate-treated mouse embryonic stem cells showed dramatic erasure of 5mC marks (32–40% decrease) over 3 days of treatment (60). These studies indicate that intracellular ascorbate availability is likely to have significant implications for cell reprograming and cancer cell biology.

### Mechanism of ascorbate activity

The mechanism by which ascorbate enhances hydroxylase activity has been thought to be due to the specific reduction of enzyme-bound Fe^3+^ to Fe^2+^. Uncoupled reaction cycles may be the primary cause of iron oxidation and although these represented only 0.7 or 1.25% of total C-P4H activity, this was found to be sufficient to oxidize enzyme-bound iron (49, 55). This oxidation could be reversed by ascorbate (51, 53, 55). Ascorbate is not stoichiometrically consumed during hydroxylase activity (54, 55), but one study found that it was stoichiometrically consumed specifically during uncoupled reaction cycles, supporting its involvement in protecting against this mode of enzyme inactivation (53). However, in another study, using recombinant PHD2, there was a significant increase in ascorbate-reversible Fe^3+^ only when the prime substrate was added, suggesting coupled turnover may also oxidize the iron (61).

We have shown that ascorbate was able to prevent induction of HIF-1α by Co^2+^, Ni^2+^, or iron chelation by DFO (62). DFO specifically chelates Fe^3+^ (63), which is formed during the hydroxylase reaction cycle, and the prevention of which suggests that ascorbate may prevent free loss of enzyme-bound Fe^3+^ or its substitution with Co^2+^ or Ni^2+^ (64). Ascorbate is known to interact with iron by reducing insoluble ferric iron complexes to stable, soluble, ferrous chelates (65, 66) and can chelate iron intracellularly (67). Together, these studies would support a specific role for ascorbate in both chelating and reducing enzyme-bound Fe^3+^ during hydroxylase reactions to maintain continued enzyme cycling.

This specificity of ascorbate for optimizing hydroxylase activity has been demonstrated; other reducing agents including glutathione, vitamin E, NADPH, dithiothreitol (DTT), L-cysteine, and tetrahydrofolic acid, were unable to substitute for ascorbate for C-P4H or TET activity (2, 54, 55, 59, 60). In addition, ascorbate was specifically necessary for full substrate hydroxylation with the HIF-hydroxylases (purified recombinant PHD2 and FIH), substantially increasing both the initial rate and extent of HIF-1α domain hydroxylation (68). Glutathione and DTT were not able to substitute for ascorbate, although DTT (68) and supra-physiological concentrations of glutathione in combination with ascorbate (69) can partially enhance PHD2 hydroxylase activity. These studies demonstrate that ascorbate is the most effective reducing agent for hydroxylase activity, with a high degree of specificity.

Ascorbate may also be structurally specific to the hydroxylase active site. There is some support for its direct binding to the active site iron in C-P4H, where it may act as an inner-sphere reductant for the iron (49, 52) and a plausible ascorbate binding site within the enzyme–substrate complex has been reported for a plant member of the 2-OGDD family, anthocyanidin synthase (70). This provides a further possible mechanism for ascorbate, where it may stabilize the enzyme–substrate interaction to facilitate hydroxylase activity. Further to this, a recent study on the AlkB protein indicates it has highly dynamic protein folding depending on whether 2-OG and Fe^2+^ are bound (71), and it has been suggested that the TET enzymes require ascorbate to assist functional protein folding to enhance catalytic activity (60). The effect of ascorbate structural analogs on PHD2 and FIH activity revealed that the enediol reducing moiety of ascorbate is essential for hydroxylase activity (68), supporting its role in maintaining reduced Fe^2+^. However, the precise binding position of ascorbate in the hydroxylase reaction cycle is still unclear.

In summary, there is a growing number of 2-OGDD family members with various cellular functions, and in those that have been studied (C-P4H, HIF-hydroxylases, and TETs), it is clear that ascorbate is required to maintain their activity. Whether other 2-OGDDs are similarly dependent on ascorbate for activity, and how that affects the relevant cellular pathways, will be of interest to investigate.

## Ascorbate Biochemistry and Pharmacokinetics

2-oxoglutarate dependent dioxygenases appear to require above 1 mM intracellular ascorbate for optimal activity, and interestingly, most cells in the body will accumulate these levels under normal, healthy physiological conditions. In most organisms, ascorbate is synthesized from glucose. Animals produce ascorbate in the liver or kidneys, from which it is transported to the plasma for distribution to the rest of the body (72). However, primates, including humans, as well as guinea pigs and some species of bat are unable to synthesize it due to evolutionary loss of the terminal synthetic enzyme, gulonolactone oxidase (72). Therefore, ascorbate is an essential nutrient (vitamin C) that we must obtain from our diet.

**Figure 2:**
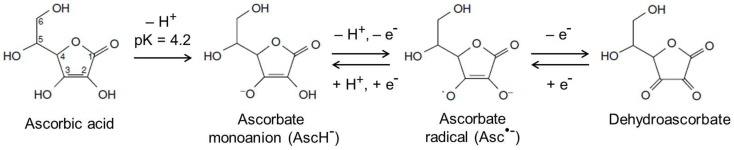
**Chemical structures of ascorbate and its oxidation products**. At physiological pH, ascorbate exists as the ascorbate monoanion and can undergo two consecutive, reversible, one-electron oxidations to produce the ascorbate radical and dehydroascorbate, respectively. Adapted from Kall (73).

Many of the known functions of ascorbate are attributable to its action as an electron donor. Ascorbate has two ionizable hydroxyl groups with pK_a_ values of 4.2 and 11.6, meaning at physiological pH, it is present as the ascorbate monoanion (Figure 2) (73). It readily undergoes two consecutive, reversible, one-electron oxidations, resulting in the ascorbate radical and dehydroascorbate (DHA; Figure 2) (73). Ascorbate is an excellent antioxidant, both thermodynamically and kinetically, is able to neutralize many highly reactive oxidizing species, and is therefore known as a terminal small molecule antioxidant (74).

Tissue ascorbate levels vary significantly and this is generally thought to reflect a functional requirement. The highest levels are found in the adrenal medulla where noradrenaline is synthesized and in the pituitary gland where many hormones are produced (75). The brain also has high levels and is the last organ to be depleted during deficiency (76), and many other tissues, including white blood cells, contain high concentrations. Tissue ascorbate levels are maintained by specific active transporters identified as sodium-dependent vitamin C transporters (SVCTs), with two known isoforms – SVCT1 that is specifically involved in gastrointestinal absorption and renal reabsorption, and SVCT2 that expressed in most tissues and is thought to be responsible for whole body cellular uptake (77, 78).

Plasma ascorbate saturation from gastrointestinal absorption is ~100 μM and the SVCTs transport it against a concentration gradient resulting in millimolar intracellular concentrations (79–81). Final intracellular ascorbate levels depend on the circulating plasma concentration available to the cells for uptake. The apparent *K_m_* values for the SVCTs have been determined in various cultured human cell lines, and range between 65 and 237 μM for SVCT1 and 8–62 μM for SVCT2, the primary mediator of intracellular uptake (82). An optimal plasma level to achieve tissue saturation is ~70–80 μM, which corresponds to a dietary intake of ~200 mg per day and tissue accumulation becomes significantly impaired if plasma levels fall below ~20 μM (83). This suggests that access to concentrations in the range of those found in plasma will substantially affect intracellular levels. Interestingly, several studies have shown that cancer patients have significantly lower plasma ascorbate levels compared to healthy controls (84–89) and this may limit tumor cell uptake.

## Ascorbate in Tumor Tissue

The ascorbate content of tumor tissue has been measured in some studies as early as the 1970s, with variable results. Brain and colorectal tumors contained significantly less ascorbate than normal tissue (90–92) whereas breast (93), oral (87), skin (94), and lung (84) cancers had significantly more ascorbate than corresponding normal tissues.

More recent studies from our lab have shown that both endometrial and colorectal tumors of high histological grade had less ascorbate than matched, adjacent normal tissue (95, 96). This may be due to a disorganized vessel network common in high grade tumors, or may reflect a reduction in expression or activity of SVCT2, which is known to vary among cancer cell lines (97), but to our knowledge, has not been examined in human cancer tissue.

Given the difficulty of accessing the plasma supply, it is possible that delivery of ascorbate to tumor cells may be compromised. Plasma levels are tightly controlled by the SVCTs, limiting absorption and reabsorption at both the intestine and kidney and do not normally exceed ~100 μM with dietary intake (83). Intravenous administration of ascorbate bypasses this tight control and can yield plasma levels up to 100-fold higher with maximum levels of up to 15 mM (98). Whether these supra-physiological concentrations would significantly increase delivery to tumor cells or cellular uptake is unknown, but investigation of this possibility may be of particular relevance to cancer.

## Potential 2-OGDD-Mediated Effects of Ascorbate on Tumor Biology

### Ascorbate and HIF-1 regulation

Several *in vitro* studies have shown that ascorbate (25–1000 μM in culture medium) can suppress HIF-1α protein stabilization and transcriptional activity due to CoCl_2_ (62, 99, 100), DFO (62, 100), and insulin-like growth factor or insulin (100). The response to hypoxia is less clear, with some of these studies reporting that ascorbate could inhibit HIF-1 at 1–3% O_2_ (62, 99–101), while other results showed no effect of ascorbate at ≤1% O_2_ (36, 100). Ascorbate was also able to completely block basal HIF-1α present in oncogenically activated cells that had mutant p53 and PTEN, indicating that it is able to enhance HIF-hydroxylase catalytic capacity in order to cope with the increase in HIF-1α synthesis (100).

One *in vivo* study has measured xenograft growth in mice of P493 cells that constitutively expressed a mutant, stabilized form of HIF-1α (102). Oral ascorbate supplementation of the animals resulted in significant inhibition of wild-type tumor growth, with no effect on mutant HIF-1α tumors (102). Interestingly, the mice in this study were able to synthesize their own ascorbate, and the control group also had reduced tumor growth compared to the mutant HIF-1α group (102). This indicates that ascorbate can inhibit HIF-1-mediated tumor growth in mice, and that higher concentrations have a greater effect. However, the plasma and tissue concentrations were not reported. To our knowledge, this is the only animal study to determine the HIF-1-dependent effects of ascorbate on tumor growth, and more data measuring HIF-1 and tumor ascorbate levels in a similar model would be particularly valuable.

We have correlated tumor ascorbate content with HIF-1 activation in human tumor tissue and, to our knowledge, these are the only studies to date to have done so. Strikingly similar patterns were seen in endometrial and colorectal cancer, with a significant inverse relationship between tumor ascorbate levels and HIF-1 activation (95, 96). We also measured disease-free survival in the colorectal cancer cohort and found that patients who had low tumor ascorbate content had shorter disease-free survival (95). This relationship was seen in a relatively small group of 50 patients, and was independent of tumor grade and stage, indicating that ascorbate may play a significant role in curbing tumor progression. Interestingly, the ascorbate content of adjacent normal tissue was not related to disease-free survival, suggesting that it is the ascorbate content of the tumor itself that is important (95). The precise mechanism behind this observation requires further clinical investigation including measurement of various 2-OGDD-regulated pathways in relation to changes in tumor ascorbate content.

### Ascorbate and epigenetic reprograming

The regulation of HIF-1 is likely to contribute to the anti-tumor activity of ascorbate (102). However, the recent discovery of the dependence of the TET enzymes on ascorbate for activity indicates that it may also act to epigenetically reprogram cancer cells. Ascorbate was observed to enhance generation of mouse and human pluripotent stem cells (103). Subsequently, the histone demethylase Jmjd1 was found to be involved in the ascorbate-dependent demethylation of pluripotency genes (104), and furthermore, modulation of TET activity by ascorbate has been implicated in somatic cell reprograming (57, 58). This has become a highly complex field, with dynamic interplay between TET expression levels and ascorbate availability (58), and also TET expression and HIF-1 activity (30). This raises interesting questions about how ascorbate levels in cancer cells could affect the epigenetic phenotype. In addition, as hypoxia may be coincident with ascorbate-deficiency, this could alter the response significantly.

The JHDM and AlkB sub-families of 2-OGDDs are also potential regulators of these processes, and it is unknown how many of them are affected by changes in availability of co-factors. Ascorbate would seem to have broad range of potential targets in a cancer cell, many of which may be currently uncharacterized, where the overall effect could “tip the balance” of signals and push cancer cells toward cell death.

## Ascorbate and Cancer

Ascorbate has a controversial history in relation to cancer treatment. In the 1970s, Ewan Cameron and Linus Pauling used both intravenous and oral ascorbate to treat 100 advanced cancer patients and found significant improvements in survival time and quality of life (105, 106). Subsequently, the Mayo Clinic sought to further investigate their findings by performing randomized, double-blind, placebo-controlled studies, using only oral ascorbate, initially with 60 advanced cancer patients who had received prior chemotherapy (107), or later in 100 colorectal cancer patients who had not received prior treatment (108). These clinical trials found no difference between ascorbate and the placebo, and ascorbate as a cancer therapy was effectively dismissed.

The recent discovery that intravenous ascorbate administration can provide 100-fold higher plasma levels compared to oral intake has renewed interest in its potential in treating cancer (98). Four credible case studies (including prostate cancer, renal cell carcinoma, bladder cancer, and B-cell lymphoma) have been reported showing intravenous ascorbate had substantial anti-cancer activity in these advanced cancer patients (109, 110), three of which were evaluated in accordance with the National Cancer Institute Best Case Series guidelines (110). However two recent studies, both with 24 patients and no control population, have shown no objective response, with the exception of three patients who had stable disease (111, 112). None of these studies have monitored tissue ascorbate levels or any biological markers other than toxicity parameters, and have been performed in an unselected patient population. Nevertheless, studies have found that high-dose ascorbate is remarkably non-toxic and well-tolerated in patients with normal renal function (111–113), a rare and valuable trait in a potential cancer therapy. This, together with evidence that some cancer patients may benefit from ascorbate treatment, should be grounds for further rigorous clinical investigations, based on sound hypotheses.

The renewed interest for a role for ascorbate in cancer has generated a growing body of *in vitro* and animal studies to determine its effectiveness against cancer cell survival and tumor growth, particularly using high millimolar doses (79). One hypothesis to support the use of high-dose ascorbate is the finding that at these pharmacological concentrations, it may act as a prodrug to deliver extracellular H_2_O_2_ that is selectively toxic to cancer cells (114, 115).

**Figure 3:**
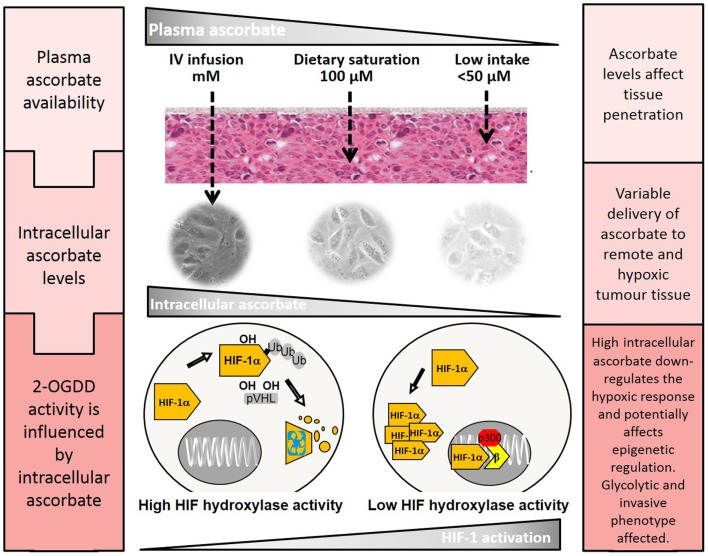
**The effect of plasma ascorbate availability on delivery to remote tumor tissue and activity of the HIF-hydroxylases**. Higher plasma ascorbate results in increased penetration of remote and hypoxic regions and the ability to down-regulate HIF-1 by promotion of the regulatory hydroxylases. HIF-1 mediated changes that regulate the tumor glycolytic phenotype, cell survival pathways, and angiogenesis could be affected, decreasing tumor viability and improving treatment outcomes. The epigenetic demethylases that also belong to the 2-OGDD family are also likely to be affected but little is known about these processes in cancer cells.

Recent animal studies have investigated the effect of ascorbate and tumor growth, all using different dosing regimens and tumor models (116–120). However, they have consistently shown an anti-tumor effect of ascorbate supplementation in mice. Mouse studies that used pharmacological ascorbate dosing (intravenous or intraperitoneal) showed a reduction in tumor growth rate and volume (116, 119–121). Other studies have used the *Gulo*−/− mouse model, in which the animals cannot synthesize ascorbate, to investigate the effect of physiological ascorbate levels (117, 118), and have also shown inhibition of tumor growth in orally ascorbate-supplemented mice. A very recent *Gulo*−/− study showed that physiological ascorbate supplementation dramatically reduced tumor metastases and necrosis, features that were associated with MMP-9 (a HIF-1 target gene) expression and tumor invasiveness (122). Serum VEGF levels were also markedly reduced (122). These mouse studies support a role for ascorbate in inhibiting tumor progression, and although the mechanisms behind some of these effects may need further clarification, the inhibition of HIF-1 is likely to contribute (102) (Figure 3).

The promise of ascorbate in treating cancer may lie in its combined use with other chemo-therapeutics. HIF-1 is known to be a driver of both chemo- and radio-resistance (123) and boosting intracellular ascorbate levels in the tumor may inhibit this effect and enhance the effectiveness of current treatments. There has been some concern that ascorbate, which is a versatile antioxidant, may in fact counteract the oxidative damage against cancer cells caused by some current cancer therapies, thereby limiting their effectiveness (124). However, recent *in vitro* and *in vivo* studies have shown that ascorbate can, in fact, enhance the effectiveness of chemotherapy (120, 125, 126). Pre-treatment of prostate cancer cells with physiological ascorbate concentrations significantly reduced the IC_50_ values of docetaxel and 5-fluorouracil (126) and pharmacological dosing of ascorbate in mice synergized with gemcitabine resulting in significantly inhibited tumor growth (125). In addition, a recent phase I clinical trial of high-dose intravenous ascorbate with gemcitabine and erlotinib in advanced pancreatic cancer has shown no adverse effects of including ascorbate (127). To our knowledge, no studies have examined the effect of intracellular ascorbate on HIF-1-induced treatment resistance. It would be of interest to study the response of tumor cells with an active HIF-1 response to a range of chemo-therapeutics, and monitor drug effectiveness in ascorbate-deficient or pre-loaded cells.

## Conclusion

The control of the 2-OGDDs by ascorbate has the potential to impact tumor growth at all stages of tumor progression. Having a high tissue ascorbate level could help prevent formation of solid tumors, slow tumor growth rates, inhibit aggressive tumor behavior, and even aid in the treatment of established cancers. Despite growing interest in ascorbate as a cancer treatment, there remains a great deal of controversy over its clinical use. However, an objective evaluation of data obtained from systematic inquiry, with an understanding of the underlying mechanisms would provide valuable insights to inform the debate. The studies summarized here clearly indicate that further investigation of the use of ascorbate in enhancing 2-OGDD activity and combating tumor progression is warranted.

## Conflict of Interest Statement

The authors declare that the research was conducted in the absence of any commercial or financial relationships that could be construed as a potential conflict of interest.
